# Supplementary material for: The singularity response reveals entrainment properties of the plant circadian clock
Source: Nat Commun. 2021 Feb 8;12:864. doi: 10.1038/s41467-021-21167-7 (PMC7870946; doi:10.1038/s41467-021-21167-7)
Supplement: Supplementary file 1 — Supplementary Information [file 41467_2021_21167_MOESM1_ESM.pdf]

Supplementary Information for "The singularity response reveals entrainment properties of the plant circadian clock"

Kosaku Masuda, Isao T. Tokuda, Norihito Nakamichi, Hirokazu Fukuda

Supplementary Figures 1-6
Supplementary Tables 1-4
Supplementary Methods

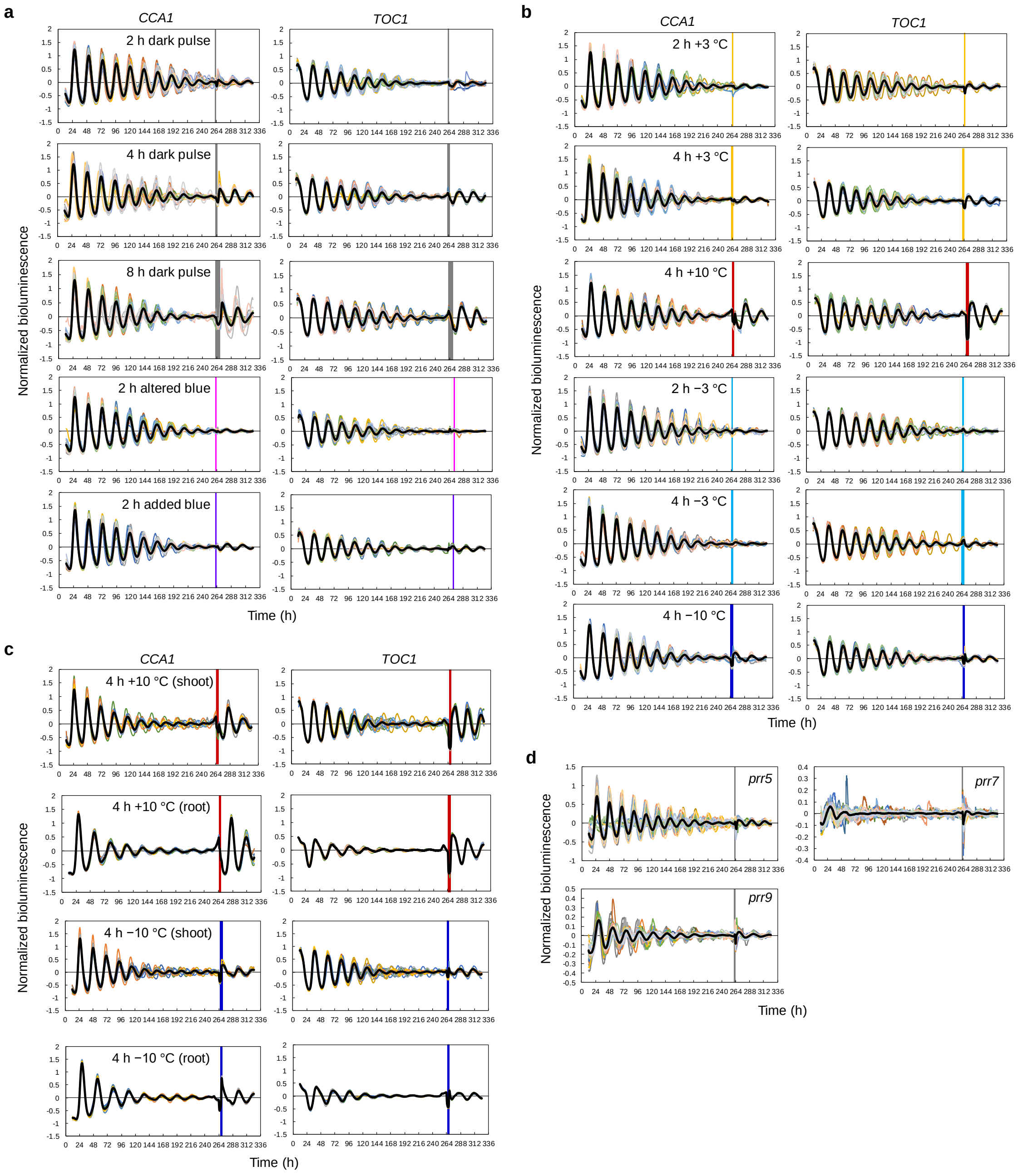

Supplementary Figure 1. Normalized bioluminescence from SR experiments.
a Dark or light stimuli. **b** Temperature stimuli. **c** Separated organs. **d** Mutant plants. The black line indicates the mean bioluminescence signal, while the colored lines represents individual bioluminescence signals.

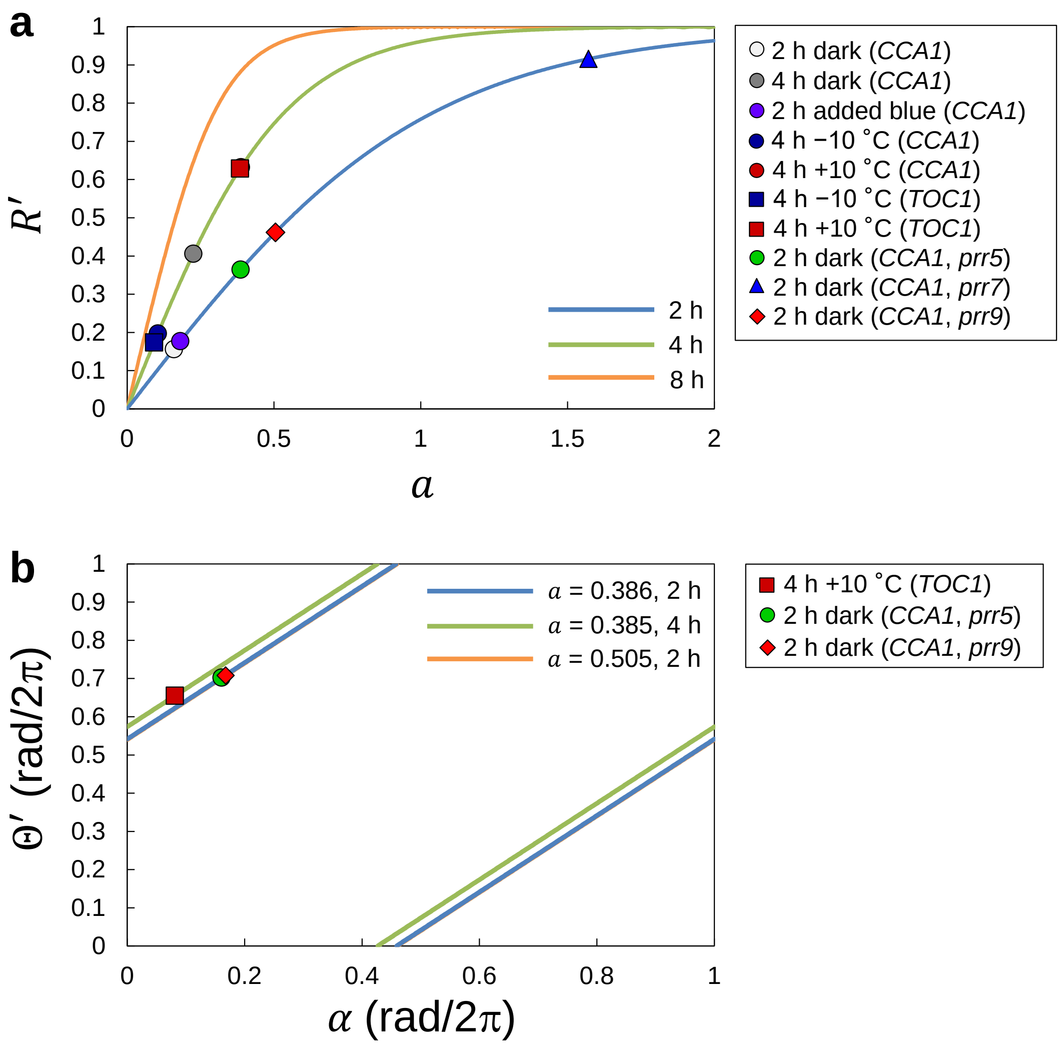

Supplementary Figure 2. Relationships between R'-to-a and Θ'-to-α.

a Relationship between R' and a. **b** Relationship between Θ' and α for estimated value of a. Solid lines were calculated by the integral on the right-hand side of Eq. (10) for variable Δt (2 h, 4 h and 8 h). The points correspond to the experimentally obtained values of R' and Θ', where R' was calibrated according to Eq. (12).

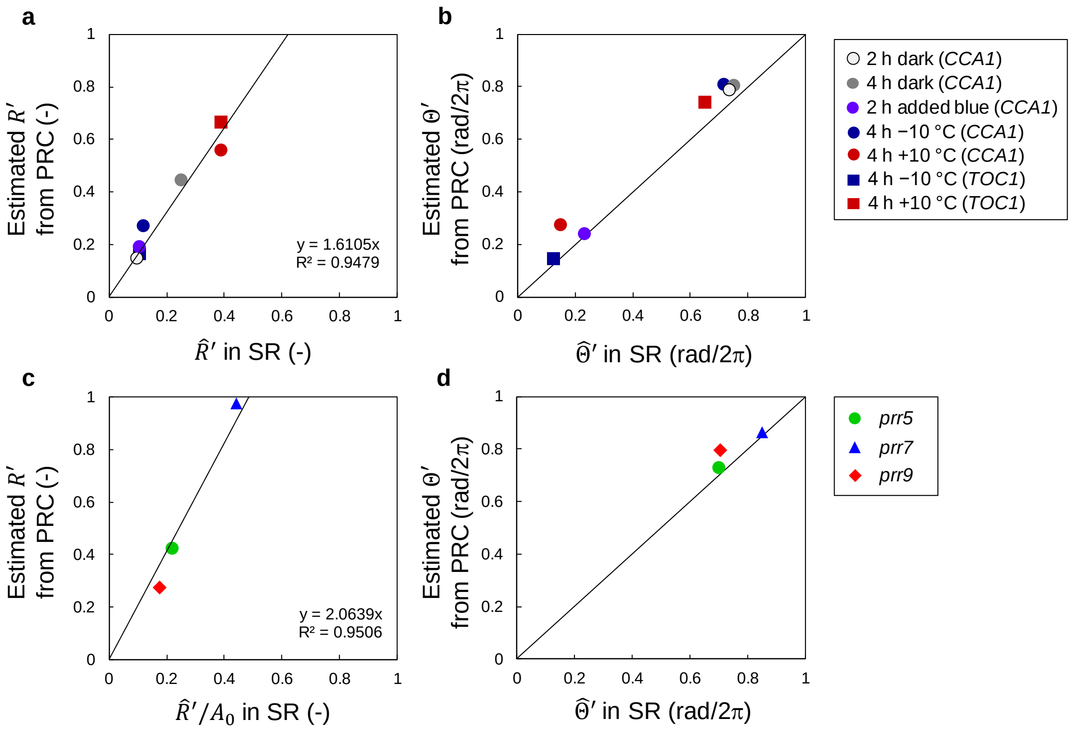

Supplementary Figure 3. Calibrations of SR from experimental data.

a $\hat{R}'$ obtained from the SR *v.s.* R' calculated from the PRC. **b** $\hat{\Theta}'$ obtained from the SR *v.s.* Θ' calculated from the PRC. **c** $\hat{R}'/A_0$ obtained from the SR *v.s.* R' calculated from the PRC, where 2 h dark is applied to *PRR* mutants. **d** $\hat{\Theta}'$ obtained from the SR *v.s.* Θ' calculated from the PRC, where 2 h dark is applied to *PRR* mutants. $\hat{R}'$ and $\hat{\Theta}'$ were calculated using Eqs. (4) and (5); R' and Θ' were calculated using Eq. (10) with the approximated curves for the PRCs measured by the standard methods. The approximated curves were given by $g(\phi) = c_1 + c_2 \sin(\phi - c_3) + c_4 \sin(2\phi - c_5)$, each parameter of which was determined by maximization of the sum of $\cos(\Delta\phi - g(\phi))$ by the generalized reduced gradient method, where ϕ and $\Delta\phi$ are the phase and phase shift, respectively, in the experimental data. For *prr7*, the approximated curve was given by $g(\phi) = c_1 + c_2 \sin(\phi - c_3) + c_4 \sin(2\phi - c_5) - \phi$, which represents a type-0 PRC, instead.

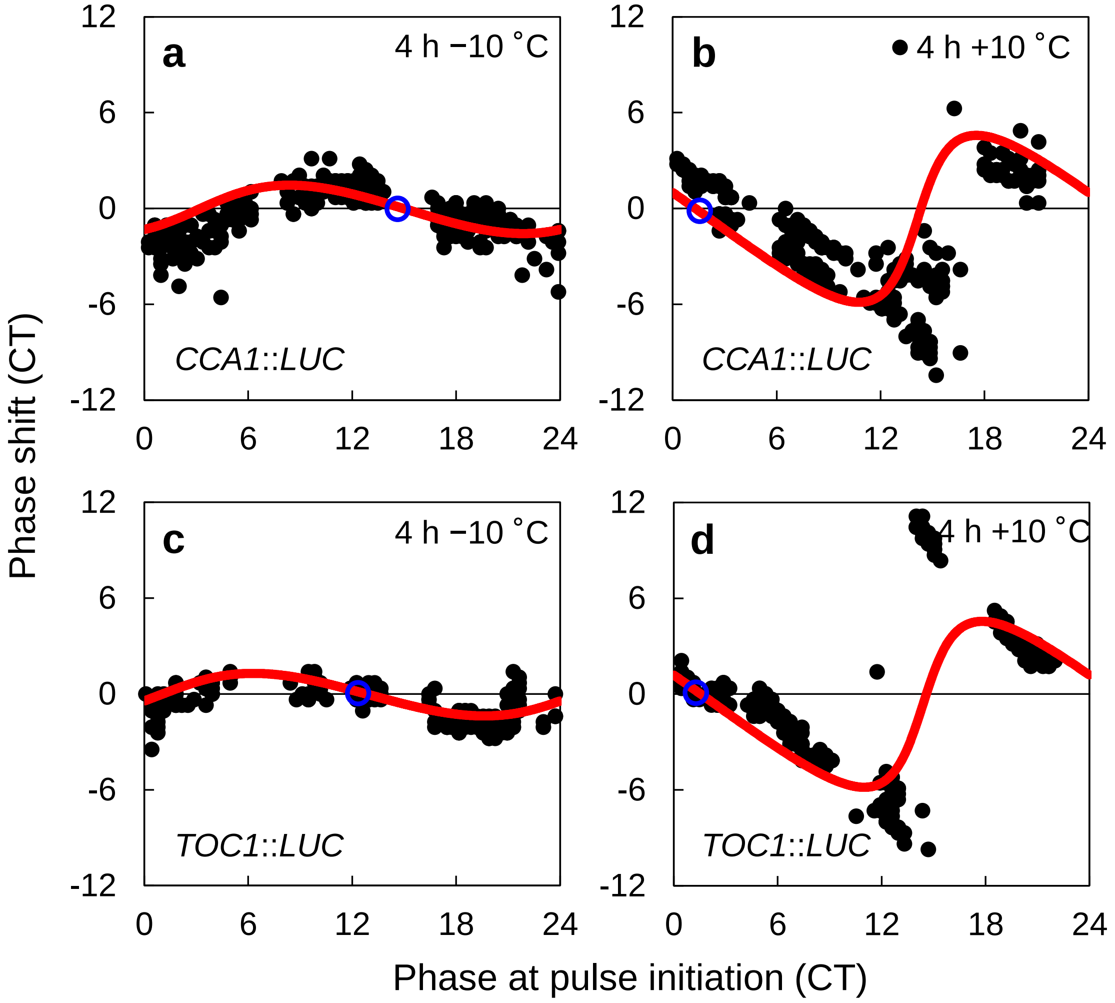

Supplementary Figure 4. PRCs for cooling and heating stimuli in *CCA1*::*LUC* and *TOC1*::*LUC* plants.

a, b PRCs for 4 h −10 °C cooling (**a**) and 4 h +10 °C heating (**b**) in *CCA1*::*LUC* plant. **c, d** PRCs for 4 h −10 °C cooling (**c**) and 4 h +10 °C heating (**d**) in *TOC1*::*LUC* plant. The solid line represents the PRC $g(\phi)$ estimated from the SR, and the dots indicate the PRC measured by the standard method. The blue circle indicates the stable point of the PRC. Phase Θ was transformed to CT as $\mathrm{CT} = \Theta/2\pi \times 24 + 2 \mod 24$ for *CCA1*::*LUC* and $\mathrm{CT} = \Theta/2\pi \times 24 + 14 \mod 24$ for *TOC1*::*LUC*.

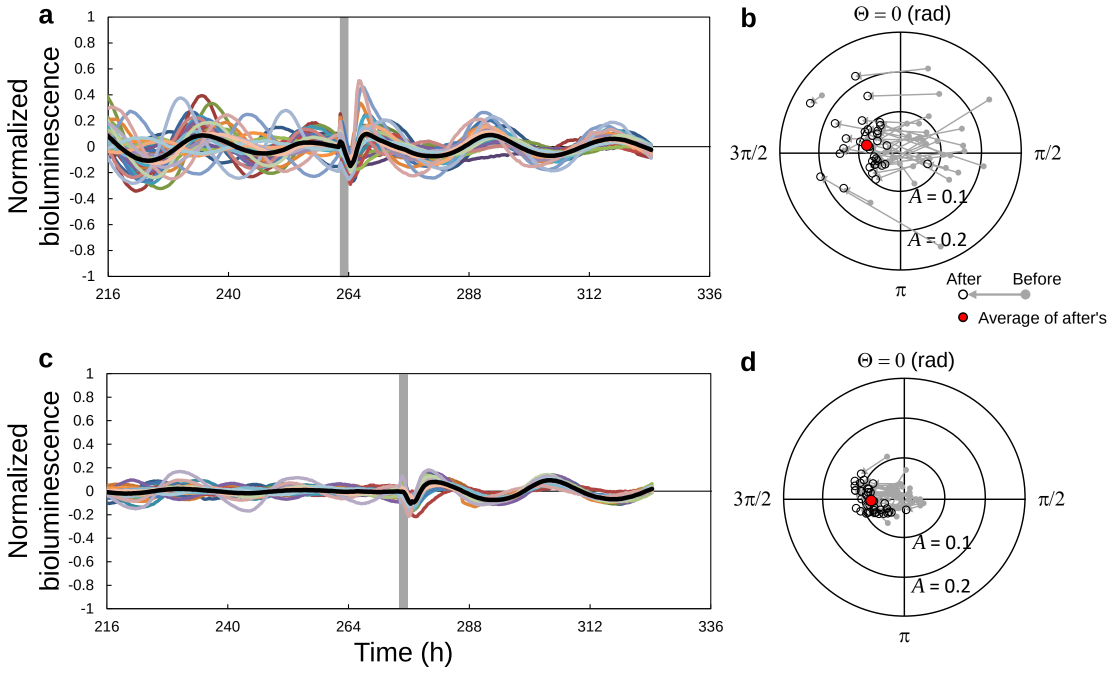

Supplementary Figure 5. Normalized bioluminescence and SR for 2-h dark pulse applied at different initial amplitude and times.

a, c Normalized bioluminescence signals of *CCA1*::*LUC* under the condition, in which dark pulse was applied at t = 262 h and t = 274 h, respectively. The black line represents the mean bioluminescence signal, while the colored lines are individual bioluminescence signals. **b, d** Phase Θ and amplitude A before and after the pulse, extracted from (**a**) and (**c**), respectively.

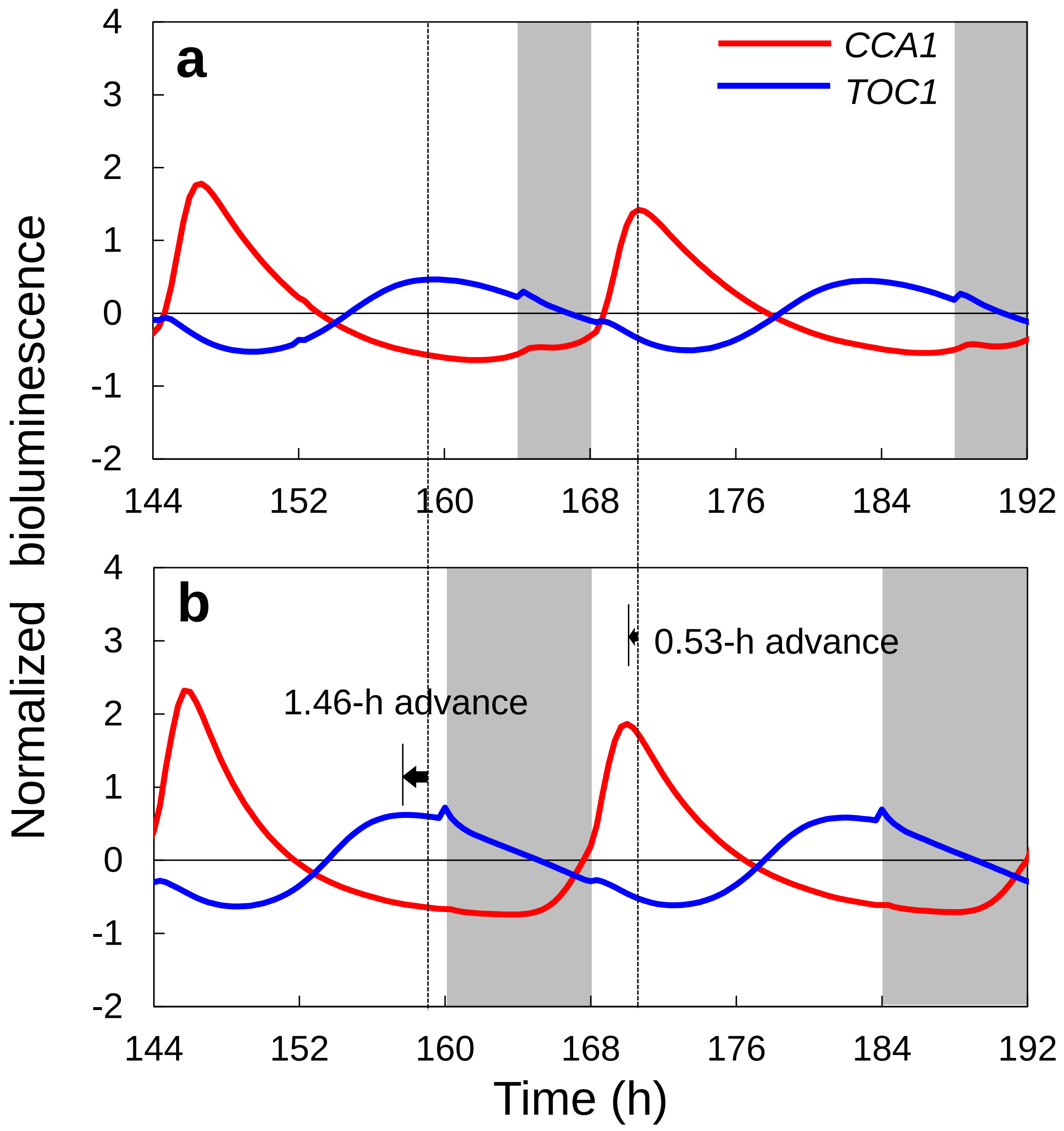

Supplementary Figure 6. Entrainments for dark cycles in *CCA1*::*LUC* and *TOC1*::*LUC* plants.

a Entrainment of an individual plant to 20:4 LD cycles. **b** Entrainment of an individual plant to 16:8 LD cycles. *CCA1*::*LUC* oscillation in (**b**) shows a 0.53-h average phase advance (n = 20) compared to that in (a). *TOC1*::*LUC* oscillation in (**b**) shows a 1.46-h average phase advance (n = 20) compared to that in (**a**).

Supplementary Table 1. Environmental conditions for the SR experiments.

Stimulus	Constant condition		Stimulus condition	
	Light (μmol m^{-2} s^{-1})	Temperature (°C)	Light (μmol m^{-2} s^{-1})	Temperature (°C)
2 h dark	Red 80, Blue 20	25	Red 0, Blue 0	25
4 h dark	Red 80, Blue 20	25	Red 0, Blue 0	25
8 h dark	Red 80, Blue 20	25	Red 0, Blue 0	25
2 h altered blue	Red 80, Blue 0	25	Red 0, Blue 80	25
2 h added blue	Red 80, Blue 0	25	Red 80, Blue 80	25
2 h +3 °C	Red 80, Blue 20	25	Red 80, Blue 20	28
2 h −3 °C	Red 80, Blue 20	25	Red 80, Blue 20	22
4 h +3 °C	Red 80, Blue 20	25	Red 80, Blue 20	28
4 h −3 °C	Red 80, Blue 20	25	Red 80, Blue 20	22
4 h +10 °C	Red 80, Blue 20	25	Red 80, Blue 20	35
4 h −10°C	Red 80, Blue 20	25	Red 80, Blue 20	15

Supplementary Table 2. Values of R' and Θ' for each stimulus.

Stimulus	*CCA1*				*TOC1*			
	R' ± SD	Θ' (rad/2π)	Circular variance	*n*	R' ± SD	Θ' (rad/2π)	Circular variance	*n*
2 h dark	0.097 ± 0.040	0.738	0.151	72	0.105 ± 0.067	0.298	0.265	18
4 h dark	0.252 ± 0.075	0.753	0.073	19	0.207 ± 0.041	0.311	0.027	20
8 h dark	0.358 ± 0.190	0.778	0.076	20	0.373 ± 0.052	0.369	0.011	19
2 h altered blue	0.063 ± 0.024	0.286	0.361	20	0.045 ± 0.031	0.859	0.476	18
2 h added blue	0.110 ± 0.028	0.234	0.037	20	0.093 ± 0.026	0.705	0.023	20
2 h +3 °C	0.073 ± 0.019	0.181	0.126	19	0.091 ± 0.049	0.652	0.038	19
2 h −3 °C	0.066 ± 0.030	0.602	0.315	20	0.061 ± 0.037	0.082	0.445	17
4 h +3 °C	0.109 ± 0.019	0.137	0.063	20	0.124 ± 0.036	0.686	0.014	17
4 h −3 °C	0.060 ± 0.023	0.772	0.358	20	0.078 ± 0.037	0.154	0.182	17
4 h +10 °C	0.392 ± 0.083	0.153	0.021	20	0.390 ± 0.052	0.655	0.003	20
4 h −10 °C	0.122 ± 0.033	0.719	0.074	20	0.108 ± 0.029	0.129	0.255	20

Supplementary Table 3. Values of R' and Θ' in separated shoots, roots, and whole plants.

Stimulus	Organ	*CCA1*				*TOC1*			
		R' ± SD	Θ' (rad/2π)	Circular variance	*n*	R' ± SD	Θ' (rad/2π)	Circular variance	*n*
4 h +10 °C	whole	0.392 ± 0.083	0.153	0.021	20	0.390 ± 0.052	0.655	0.003	20
	shoot	0.477 ± 0.091	0.182	0.019	10	0.541 ± 0.109	0.671	0.004	10
	root	0.938 ± 0.063	0.331	0.002	10	0.452 ± 0.021	0.734	0.001	10
4 h −10 °C	whole	0.122 ± 0.033	0.719	0.074	20	0.108 ± 0.029	0.129	0.255	20
	shoot	0.153 ± 0.061	0.766	0.010	10	0.154 ± 0.056	0.121	0.259	10
	root	0.260 ± 0.047	0.855	0.002	10	0.134 ± 0.018	0.327	0.172	10

Supplementary Table 4. Values of the natural amplitude A_0, R', and Θ' in mutants.

Genotype	Period (h)	Θ' (rad/2π)	Circular variance	A_0	R'	R'/A_0	n
Col	23.18 ± 0.25	0.738	0.151	0.933 ± 0.104	0.097 ± 0.040	0.107 ± 0.055	72
prr5	21.27 ± 0.57	0.702	0.067	0.584 ± 0.163	0.096 ± 0.029	0.177 ± 0.069	27
prr7	22.85 ± 1.71	0.851	0.460	0.068 ± 0.044	0.027 ± 0.010	0.444 ± 0.170	22
prr9	23.42 ± 0.66	0.708	0.237	0.133 ± 0.045	0.028 ± 0.013	0.222 ± 0.105	50

Supplementary Methods

Analytical solution for PRC $g(\phi)$

Putting $\psi = \phi - \alpha$ in Eq. (8) under the stimulation (i.e. $E(t) = 1$), the following equation is obtained:

$$\frac{d\psi}{dt} = \omega + a \sin \psi. \tag{S1}$$

Since Eq. (S1) does not include α, its solution ψ is independent of α.

Dividing both sides of Eq. (S1) by the right-hand side yields

$$\frac{1}{\omega + a \sin \psi} \frac{d\psi}{dt} = 1. \tag{S2}$$

Integrating both sides of Eq. (S2) in $[0, \Delta t]$, we obtain

$$\int_{\psi(0)}^{\psi(\Delta t)} \frac{1}{\omega + a \sin \psi} d\psi = \Delta t. \tag{S3}$$

Putting $s = \tan\frac{\psi}{2}$, the variables are transformed as

$$\sin \psi = \frac{2s}{1 + s^2}, \tag{S4}$$

$$d\psi = \frac{2}{1 + s^2} ds, \tag{S5}$$

while the integration range is transformed as $\psi : \psi(0) \to \psi(\Delta t)$, $s : \tan\frac{\psi(0)}{2} \to \tan\frac{\psi(\Delta t)}{2}$.

Hence, the left-hand side of Eq. (S3) is described as follows:

$$\int_{\psi(0)}^{\psi(\Delta t)} \frac{1}{\omega + a \sin \psi} d\psi = \int_{\tan\frac{\psi(0)}{2}}^{\tan\frac{\psi(\Delta t)}{2}} \frac{1}{\omega + a \frac{2s}{1 + s^2}} \frac{2}{1 + s^2} ds$$

$$= \int_{\tan\frac{\psi(0)}{2}}^{\tan\frac{\psi(\Delta t)}{2}} \frac{2}{\omega(1 + s^2) + 2as} ds\,. \tag{S6}$$

Thus,

$$\int_{\tan\frac{\psi(0)}{2}}^{\tan\frac{\psi(\Delta t)}{2}} \frac{2}{\omega(1 + s^2) + 2as} ds = \Delta t. \tag{S7}$$

Depending on the relationship between ω and a, the solution of $\psi(\Delta t)$ is obtained as follows.

If $a < \omega$,

$$\psi(\Delta t) = 2\tan^{-1}\left\{\frac{\sqrt{\omega^2 - a^2}}{\omega}\tan\left(\frac{\sqrt{\omega^2 - a^2}}{2}\Delta t + \tan^{-1}\left(\frac{\omega}{\sqrt{\omega^2 - a^2}}\left(\tan\frac{\psi(0)}{2} + \frac{a}{\omega}\right)\right)\right) - \frac{a}{\omega}\right\}. \tag{S8}$$

If $a > \omega$,

$$\psi(\Delta t) = 2\tan^{-1}\left\{\frac{\frac{a + \sqrt{a^2 - \omega^2}}{a - \sqrt{a^2 - \omega^2}}e^{\sqrt{a^2-\omega^2}\Delta t} - \frac{\omega\tan\frac{\psi(0)}{2} + a + \sqrt{a^2 - \omega^2}}{\omega\tan\frac{\psi(0)}{2} + a - \sqrt{a^2 - \omega^2}}}{\frac{\omega}{a - \sqrt{a^2 - \omega^2}}\left(\frac{\omega\tan\frac{\psi(0)}{2} + a + \sqrt{a^2 - \omega^2}}{\omega\tan\frac{\psi(0)}{2} + a - \sqrt{a^2 - \omega^2}} - e^{\sqrt{a^2-\omega^2}\Delta t}\right)}\right\} \tag{S9}$$

If $a = \omega$,

$$\psi(\Delta t) = 2\tan^{-1}\left\{\frac{\tan\frac{\psi(0)}{2} + \left(\tan\frac{\psi(0)}{2} + 1\right)\omega\Delta t}{1 - \left(\tan\frac{\psi(0)}{2} + 1\right)\omega\Delta t}\right\} \tag{S10}$$

With respect to ψ, the phase response function, $g_\psi(\psi(0))$, to stimulus duration of Δt is defined as follows:

$$g_\psi(\psi(0)) = \psi(\Delta t) - \psi(0) - \omega\Delta t. \tag{S11}$$

$g_\psi(\psi)$ is independent of α because $\psi(\Delta t)$ is independent of α.

Since $\psi = \phi - \alpha$ and $g_\phi(\phi(0)) = \phi(\Delta t) - \phi(0) - \omega\Delta t$,

$$\begin{aligned} g_\phi(\phi(0)) &= (\phi(\Delta t) - \alpha) - (\phi(0) - \alpha) - \omega\Delta t \\ &= \psi(\Delta t) - \psi(0) - \omega\Delta t \\ &= g_\psi(\psi(0)) \end{aligned} \tag{S12}$$

Using the phase response function $g_\phi(\phi(0))$, the singularity response quantities, R' and Θ', are given by the following equation:

$$R'e^{i\Theta'} = \frac{1}{2\pi}\int_0^{2\pi} e^{i(\phi + g_\phi(\phi) + \omega\Delta t)}d\phi \tag{S13}$$

Since $\phi = \psi + \alpha$, $g_\phi(\phi) = g_\psi(\psi)$ and $d\phi = d\psi$,

$$R'e^{i\Theta'} = \frac{1}{2\pi}\int_{-\alpha}^{2\pi-\alpha} e^{i(\psi + \alpha + g_\psi(\psi) + \omega\Delta t)}d\psi. \tag{S14}$$

Hence,

$$R'e^{i(\Theta'-\alpha)} = \frac{1}{2\pi}\int_{-\alpha}^{2\pi-\alpha} e^{i(\psi+g_\psi(\psi)+\omega\Delta t)}d\psi. \tag{S15}$$

Because $g_\psi(\psi)$ is independent of α and the result of integration over one cycle is independent of the integration range ($[0,2\pi]$ or $[-\alpha, 2\pi-\alpha]$), the right-hand side of Eq. (S15) is independent of α and thus described as follows:

$$R'e^{i(\Theta'-\alpha)} = H(a,\Delta t)e^{iI(a,\Delta t)}. \tag{S16}$$

Therefore, the following relationship is obtained:

$$R' = H(a,\Delta t), \tag{S17}$$

$$\Theta' = \alpha + I(a,\Delta t). \tag{S18}$$

Supplementary Fig. 2 shows one-to-one correspondence between a and R' and between α and Θ'. Using these relationships, we can determine a and α such that R' and Θ' are consistent with the experimental values. Using the estimated a and α, the PRC $g(\phi)$ can be drawn by Eq. (S12).
